# Therapeutic effects of an aspalathin-rich green rooibos extract, pioglitazone and atorvastatin combination therapy in diabetic *db/db* mice

**DOI:** 10.1371/journal.pone.0251069

**Published:** 2021-05-13

**Authors:** Oelfah Patel, Christo J. F. Muller, Elizabeth Joubert, Bernd Rosenkranz, Johan Louw, Charles Awortwe

**Affiliations:** 1 Biomedical Research and Innovation Platform (BRIP), South African Medical Research Council (MRC), Tygerberg, South Africa; 2 Division of Clinical Pharmacology, Department of Medicine, Faculty of Medicine and Health Sciences, University of Stellenbosch, Tygerberg, South Africa; 3 Division of Medical Physiology, Faculty of Health Sciences, Stellenbosch University, Tygerberg, South Africa; 4 Department of Biochemistry and Microbiology, University of Zululand, KwaDlangezwa, South Africa; 5 Post-Harvest and Agro-Processing Technologies, Agricultural Research Council, Infruitec- Nietvoorbij, Stellenbosch, South Africa; 6 Department of Food Science, Stellenbosch University, Matieland, South Africa; 7 Institute of Experimental and Clinical Pharmacology, University Hospital Schleswig-Holstein, Kiel, Germany; The Ohio State University College of Medicine, UNITED STATES

## Abstract

Oral therapeutics used to treat type 2 diabetes and cardiovascular disease often fail to prevent the progression of disease and their comorbidities. Rooibos (*Aspalathus linearis*), an endemic South African plant used as an herbal tea, has demonstrated positive effects on glycemia and hypercholesterolemia. However, the treatment efficacy of rooibos extract in combination with conventional hypoglycemic and hypolipidemic medications on blood glucose and lipid profiles has not been established. This study aimed to investigate the effects of combining an aspalathin-rich green rooibos extract (Afriplex GRT^™^) with pioglitazone and atorvastatin, on blood glucose and lipid levels in obese diabetic (*db/db*) mice. Six-week-old male *db/db* mice and their nondiabetic lean littermate controls (*db+*) were divided into 8 experimental groups (n = 6/group). *Db/db* mice were treated daily either with pioglitazone (25 mg/kg), atorvastatin (80 mg/kg) and GRT (100 mg/kg), a combination of either drug with GRT or a combination of GRT-pioglitazone and atorvastatin for 5 weeks. Untreated vehicle controls were given dimethyl sulfoxide (0.1%) and phosphate buffered saline solution. At termination, serum and liver tissue were collected for lipid and gene expression analysis. Treatment with GRT, pioglitazone and atorvastatin combination effectively lowered fasting plasma glucose (FPG) levels in *db/db* mice (p = 0.02), whilst increasing body weight, liver weight, and reducing retroperitoneal fat weight. Atorvastatin monotherapy was effective at reducing cholesterol (from 4.00 ± 0.12 to 2.93 ± 0.13, p = 0.0003), LDL-C (from 0.58 ± 0.04 to 0.50 ± 0.00, p = 0.04), HDL-C (from 2.86 ± 0.05 to 2.50 ± 0.04, p = 0.0003) and TG (from 2.77 ± 0.50 to 1.48 ± 0.23, p = 0.04), compared to the untreated diabetic control. The hypotriglyceridemic effect of atorvastatin was enhanced when used in combination with both GRT and pioglitazone. The addition of pioglitazone to GRT significantly lowered FPG and TG. In *db/db* mice, *Apoa1* was significantly downregulated in the liver, whilst *Pparγ* was significantly upregulated compared to their *db+* counterparts. GRT monotherapy downregulated *Apoa1* expression (p = 0.02). Atorvastatin combined with GRT significantly downregulated mRNA expression of *Apoa1* (p = 0.03), whilst upregulating the expression of *Pparγ* (p = 0.03), *Pparα* (p = 0.002), *Srebp1* (p = 0.002), and *Fasn* (p = 0.04). The GRT-pioglitazone-atorvastatin combination therapy downregulated *Apoa1* (p = 0.006), whilst upregulating *Fasn* (p = 0.005), *Pparα* (p = 0.041), and *Srebp1* (p = 0.03). Natural products can improve the efficacy of current drugs to prevent diabetes-associated complications. GRT in combination with pioglitazone enhanced the reduction of FPG, whilst the addition of atorvastatin to the combination, significantly lowered triglyceride levels. However, when GRT was used in combination with atorvastatin only cholesterol levels were affected. Although these results confirm both glucose- and lipoprotein-lowering biological effects of GRT in combination with pioglitazone and atorvastatin, increased expression of genes involved in lipogenesis, cholesterol, and fatty acid transport, β-oxidation, and synthesis and storage of fatty acids, may exacerbate the hepatotoxic effects of atorvastatin.

## Introduction

Metabolic conditions such as insulin resistance, dyslipidemia and increased oxidative stress underlie the development of diabetes and cardiovascular disease [1, 2]. In diabetics, hyperglycemia and dyslipidemia increases the risk of atherosclerosis, impaired cardiac function, and adverse cardiovascular events such as myocardial infarction and heart failure [3–6]. Treating the clinical complexity of hyperglycemia and dyslipidemia generally requires combination therapy [7, 8].

Pioglitazone, a thiazolidinedione derivative, is an oral glucose-lowering medication commonly prescribed to treat type 2 diabetes (T2D). Peroxisome proliferator-activated receptors (PPARs) are key transcriptional regulators of lipid and carbohydrate metabolism, energy production and regulating cardiovascular function [9]. Pioglitazone is a high-affinity ligand of PPARγ that activates and translocates PPARγ to the nucleus. Once in the nucleus it forms a complex with the retinoid x receptor alpha in modulating gluconeogenic genes in the liver and induces transcription of the insulin-sensitive glucose transporter, GLUT4 in adipose tissue [10–12], enhancing glucose transport and utilization, as well as suppressing hepatic glucose production [13, 14]. Pioglitazone is classified as a weak ligand for PPARα, and as such reduces insulin resistance, suppresses inflammation, and infers a role in increasing β-oxidation [15]; whilst improving hepatic steatosis and non-alcoholic fatty liver disease [16].

Atorvastatin belongs to the statin class of competitive inhibitors of 3-hydroxy-3-methyl-glutaryl coenzyme-A (HMG-CoA) reductase and is the most prescribed cholesterol-lowering agent for treating elevated low-density lipoprotein-cholesterol (LDL-C), triglycerides (TGs), and cholesterol levels [17–19]. Statins act by inhibiting HMG-CoA reductase, increasing the expression of LDL receptors on the hepatocytes and the uptake of LDL-C from the plasma into the liver [20]. Statins also inhibit Rho-kinase, a key enzyme activating the pro-atherogenic Rho/ROCK pathway, eliciting a cardiovascular protective effect independent of its LDL-C lowering effect [21, 22]. In addition, atorvastatin is considered a dual activator of PPARγ and PPARα, lowering pro-inflammatory cytokines such as TNF-α and triglycerides [9]. Although statins have a cardioprotective role, they may cause adverse effects such as myopathy, rhabdomyolysis, and liver injury.

By combining atorvastatin with pioglitazone, a reduction in LDL-C and triglyceride levels as well as atherogenic processes by atorvastatin, together with the insulin-sensitizing effects of pioglitazone, provides a favorable therapeutic regime [23]. In addition to these conventional medications, patients presenting with glucose intolerance and dyslipidemia are progressively considering natural products as an alternative or adjunctive therapy due to their perceived added health benefits and efficacy [24]. Several of these natural products contain phenolic compounds such as flavonoids known to be largely responsible for the biological activity, including beneficial effects on glucose and lipid metabolism [25]. Within this context, several studies have alluded to the glucose and lipid modulating effects of *Aspalathus linearis* (rooibos) [26–29], a plant better known for its use as an herbal tea. The Aspalathus genus uniquely contains a C-glucosyl dihydrochalcone, aspalathin [29], which was demonstrated to stimulate glucose uptake in muscle tissues and increases insulin release in pancreatic beta cells, thus benefiting glucose homeostasis in various models of T2D [30]. In addition, rooibos has also been shown to have LDL-C lowering [31, 32], and cardioprotective effects [33–36]. In this study, we assessed the safety and pharmacological effects of an aspalathin-rich green rooibos extract (Afriplex GRT^™^) in combination with pioglitazone and atorvastatin in a diabetic *db/db* mouse model.

## Materials and methods

### Materials

Pioglitazone hydrochloride (PHG 1632, Lot # LRAA5299) and atorvastatin calcium (PHR1422, Lot # LRAA9204) were purchased from Sigma-Aldrich (St Louis, MO, USA).

### Extract preparation

A pharmaceutical certified grade, aspalathin-rich, green rooibos (Afriplex GRT^™^) extract, containing 12.8% aspalathin, was obtained from Afriplex Pharmaceuticals PTY (LTD) (Paarl, SA). Quantification of aspalathin and other flavonoids present in the extract was previously described by Patel *et al*. (2016) [37]. GRT was dissolved in 0.1% DMSO (Sigma- Aldrich, St Louis, MO, USA) and Dulbecco’s phosphate buffered saline (Lonza, Walkersville, MD, USA).

### Animals and diet

Six-week-old male *db/db* mice were bred and housed at the Primate Unit Delft Animal Centre (PUDAC) under 24 light/dark cycle in a temperature-controlled room with food and water ad libitum. The Ethics Committee for Research on Animals (ECRA) of the South African Medical Research Council (SAMRC) approved all procedures involving the animals in this study (ECRA Approval No. 04/15). Mice were divided into 8 experimental groups (n = 6 mice per group), receiving GRT (100 mg/kg BW), pioglitazone (25 mg/kg BW), and atorvastatin (80 mg/kg BW) as mono-, co-therapies of GRT and atorvastatin, GRT and pioglitazone, and the combination of GRT, atorvastatin and pioglitazone. *Db/db* and *db+* controls received 0.1% DMSO and Dulbecco’s phosphate buffered saline. Mice were treated by oral gavage once daily for 5 weeks. Body weight and fasting blood glucose (FPG) measurements via tail prick and the use of a glucometer (One-Touch Select^™^, Lifespan Europe, Switzerland) were performed weekly. After 3 weeks of treatment an intraperitoneal glucose tolerance test was conducted by injecting mice intraperitoneally with 0.2 g of glucose/ml/100 g body weight. At termination, mice were anesthetized by isoflurane inhalation (4–5% initiation of anesthesia, reduced to 2–3% for maintenance), and blood samples collected from the inferior vena cava. Serum was obtained for lipid analyses and liver tissues were excised, weighed, and snap frozen in liquid nitrogen for gene expression analyses and liver tissues were fixed on slides for histological assessment. No animals became ill or died during the study period.

### Histological assessment

Formalin fixed paraffin sections of liver tissue were stained with hematoxylin-eosin (H&E) and scored histologically for steatotic changes. Hepatic steatosis of the obese diabetic *db/db* mice were assessed histologically by an experienced histologist, blinded to the treatment groups, using a steatotic severity scoring system adapted from Trak-Smayra *et al*. (2011) [38] and Liang *et al*. (2014) [39]. Steatosis was assessed for hepatocellular steatosis type (microvesicular, mediovesicular and macrovesicular), grade or severity [0 < 5%, grade 1 (5–33%), grade 2 (34–66%) and grade 3 (> 66%)] and zonal predominance [periportal (zone 1), mediolobular (zone 2) or centrilobular (zone 3)]. Samples were blind-coded and randomly assessed to avoid observational bias by the histologist.

### mRNA expression

Total RNA was extracted from mouse liver tissue using the RNeasy kit (ThermoFischer Scientific Inc., Waltham, MA, USA). Tissues were homogenized using a TissueLyser (Qiagen GmbH, Hilden, Germany), centrifuged at 13, 500 g for 3 min, and the extracted RNA purified using the RNeasy kit according to the manufacturer’s instructions. RNA concentration and purity were quantified using a Nanodrop One spectrophotometer (Thermo Electron Scientific Instruments LLC, Madison, WI, USA). The RNA quality was determined using an Agilent 2100 Bioanalyzer (Agilent Technologies, Santa Clara, CA, USA). A Turbo DNase kit (ThermoFischer Scientific Inc.) was used to remove genomic DNA as per the manufacturer’s recommendations. RNA samples were converted to cDNA using the High Capacity Reverse Transcription Kit (Applied Biosystems, Foster City, CA, USA) as recommended by the manufacturer. Quantitative real-time PCR was performed on the ABI 7500 Instrument (ThermoFischer Scientific Inc. Waltham, MA, USA) using the standard curve method. Predesigned and optimized TaqMan gene expression probes for *Apoa1* (Mm00437569_m1), *Fasn* (Mm00662319_m1), *Pparγ* (Mm00440940_m1), *Pparα* (Mm00440939_m1), *Scd1* (Mm00772290_m1) and *Srebp1* (Mm00550338_m1) were used for differential gene expression (ThermoFisher Scientific). Gene expression data were normalized to *β-actin* and hypoxanthine-guanine phosphoribosyltransferase (*HPRT*), respectively.

### Biochemical measurements

Blood samples were collected in BD Vacutainer^®^ SST gel tubes, centrifuged at 1792 g for 15 min at 4°C and the sera stored at -80°C until assayed. All samples were analyzed by Pathcare (Dietrich, Voigt, Mia & Partners, N1 City, Cape Town, SA), a pathology laboratory accredited by the South African National Accreditation System (SANAS). Briefly, total cholesterol was determined using the cholesterol esterase enzymatic method. A coupled enzymatic reaction using adenosine triphosphate (ATP) as an agent was used to determine the triglyceride (TG) content. A cholesterol esterase/cholesterol oxidase method, based on an enzyme chromogen system for quantification, was used to determine high-density (HDL-C) and low-density lipoprotein cholesterol (LDL-C) content, respectively.

### Statistical analysis

Statistical analyses were performed using GraphPad Prism^®^ version 7.03 (GraphPad Software Inc.). All *in vivo* and gene expression data are presented as the means ± SEM as well as means ± SD for gene expression and statistically compared using one-way ANOVA with Dunnett/Bonferroni post hoc test compared to the control when p < 0.05. Data was considered statistically different if p < 0.05.

## Results

### *Db/db* mouse diabetic parameters

**Fig 1:**
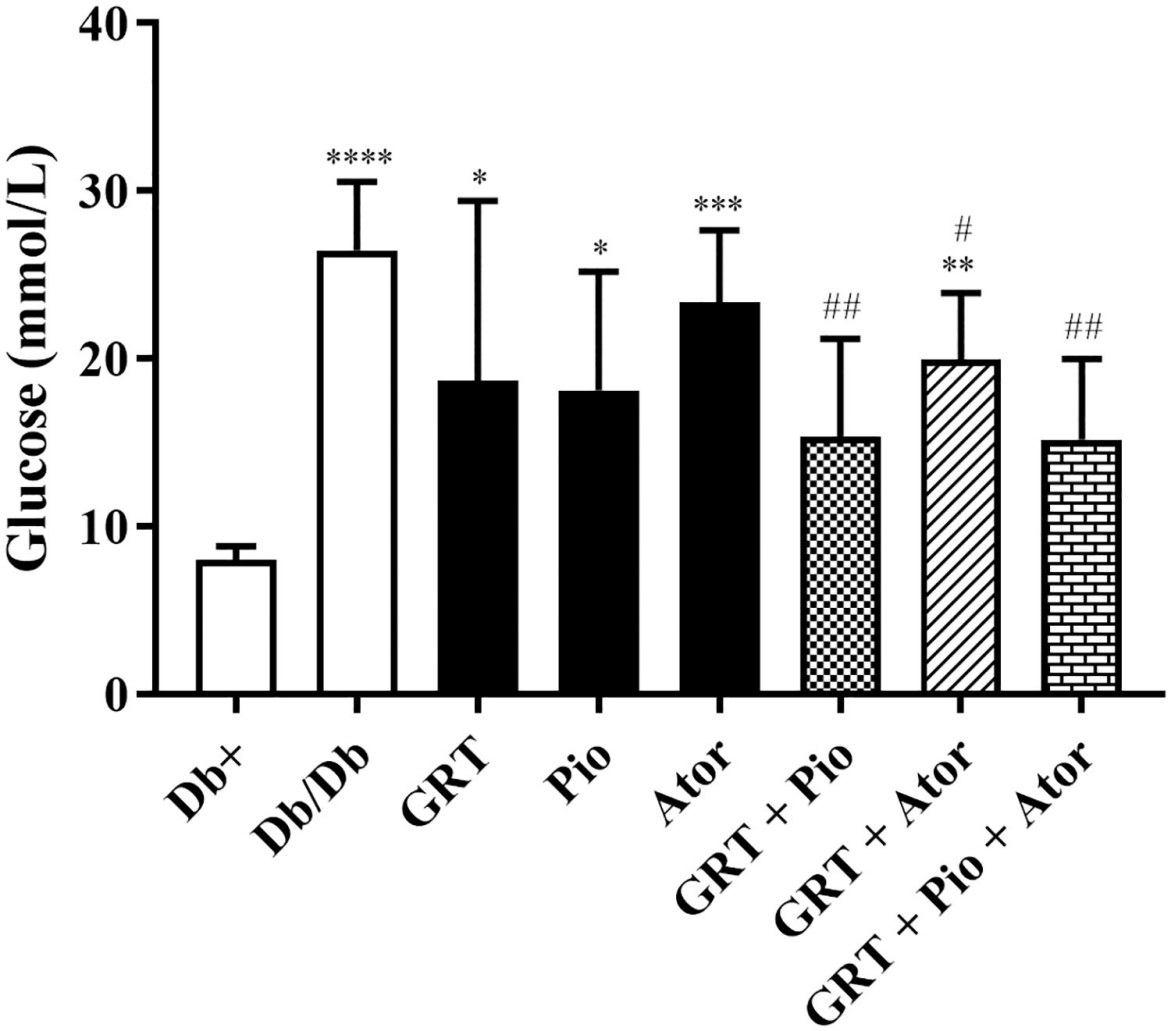
The effect of GRT (100 mg/kg BW), Pio (25 mg/kg BW), and Ator (80 mg/kg BW) mono- and co-therapies on FPG. Glucose measured after 5 weeks of treatment. Results are expressed as the mean ± SEM (n = 6). *p < 0.05, **p < 0.01 and ****p < 0.0001 vs *db+*; ^#^p < 0.05 vs *db/db* (One-Way ANOVA followed by Dunnett multiple comparison test). FPG, fasting blood glucose; GRT, green rooibos extract; Pio, pioglitazone; and Ator, atorvastatin.

At 11-weeks of age (from 6-weeks-old and 5-weeks treatment period), the obese *db/db* mice presented with elevated fasting blood glucose levels compared to their lean (*db+*) counterparts (7.98 ± 0.34 versus 26.44 ± 1.84; p < 0.0001) (Fig 1). Body weight (BW), liver weight, retroperitoneal fat weights and the liver to BW ratio of *db/db* mice were significantly increased compared to the *db+* mice (Table 1). Compared to the lean *db+* controls the *db/db* mice are hyperphagic (*db+* vs *db/db*: from 24.40 ± 0.2 to 30.20 ± 0.2). Treatments of these obese *db/db* mice did not significantly affect the average food intake during the 5-week treatment period (data not shown).

**Table 1:** Body weight and liver weight changes of mice orally treated with GRT, pioglitazone, and atorvastatin as mono- and co-therapies for 5 weeks.

	*db+*	*db/db*	GRT (100 mg/kg)	Pio (25 mg/kg)	Ator (80 mg/kg)	GRT (100 mg/kg) + Pio (25 mg/kg)	GRT (100 mg/kg) + Ator (80 mg/kg)	GRT (100 mg/kg) + Pio (25 mg/kg) + Ator (80 mg/kg)
**Body weight (g)**	25.00 ± 0.86	39.05 ± 0.23****	42.32 ± 0.82****^,^ ^#^	45.18 ± 2.46****	40.00 ± 0.67****	45.98 ± 1.18****^,^ ^##^	41.40 ± 0.72****^,^ ^#^	47.22 ± 1.50****^,^ ^##^
**Liver (g)**	1.35 ± 0.04	2.46 ± 0.07***	2.67 ± 0.13****	3.20 ± 0.30****	2.41 ± 0.08****	3.50 ± 0.22****^,^ ^##^	2.72 ± 0.04****^,^ ^#^	3.27 ± 0.19****^,^ ^#^
**RF (g)**	0.09 ± 0.01	1.35 ± 0.35***	0.67 ± 0.04*	0.57 ± 0.04	0.45 ± 0.04^#^	0.54 ± 0.04^#^	0.51 ± 0.07^#^	0.59 ± 0.05*^,^ ^#^
**Liver to BW ratio**	5.41 ± 0.06	6.28 ± 0.14***	6.30 ± 0.28	7.02 ± 0.33	6.01 ± 0.18	7.57 ± 0.34****^,^ ^##^	6.56 ± 0.16*	6.91 ± 0.28***

Results are presented as the mean ± SEM (n = 6) of body weight (g), liver weight (g), and RF weight (g).

*p < 0.05,

**p < 0.01 and

****p < 0.0001 vs *db+*;

^#^p < 0.05,

^##^p < 0.01 and

^###^p < 0.0001 vs *db/db* (One-Way ANOVA followed by Dunnett multiple comparison test). GRT, green rooibos extract; Pio, pioglitazone; Ator, atorvastatin; BW, body weight and RF, retroperitoneal fat.

### GRT, pioglitazone, and atorvastatin co-therapy improves glycemic control

After the 5-week treatment period, lower FPG levels were obtained following the co-therapy of GRT with pioglitazone (from 26.44 ± 1.84 to 15.33 ± 2.39; p = 0.006) and atorvastatin (from 26.44 ± 1.84 to 19.93 ± 1.63; p = 0.03), respectively (Fig 1). The combination of GRT-pioglitazone-atorvastatin effectively lowered the FPG level compared to the *db/db* control (from 26.44 ± 1.84 to 15.17 ± 1.96; p = 0.003). In comparison to the combination therapy, GRT, atorvastatin and pioglitazone monotherapies did not reduce FPG levels.

### GRT, pioglitazone, and atorvastatin co-therapy reduces TG, cholesterol, and LDL-C levels, and improves hepatic steatosis

**Fig 2:**
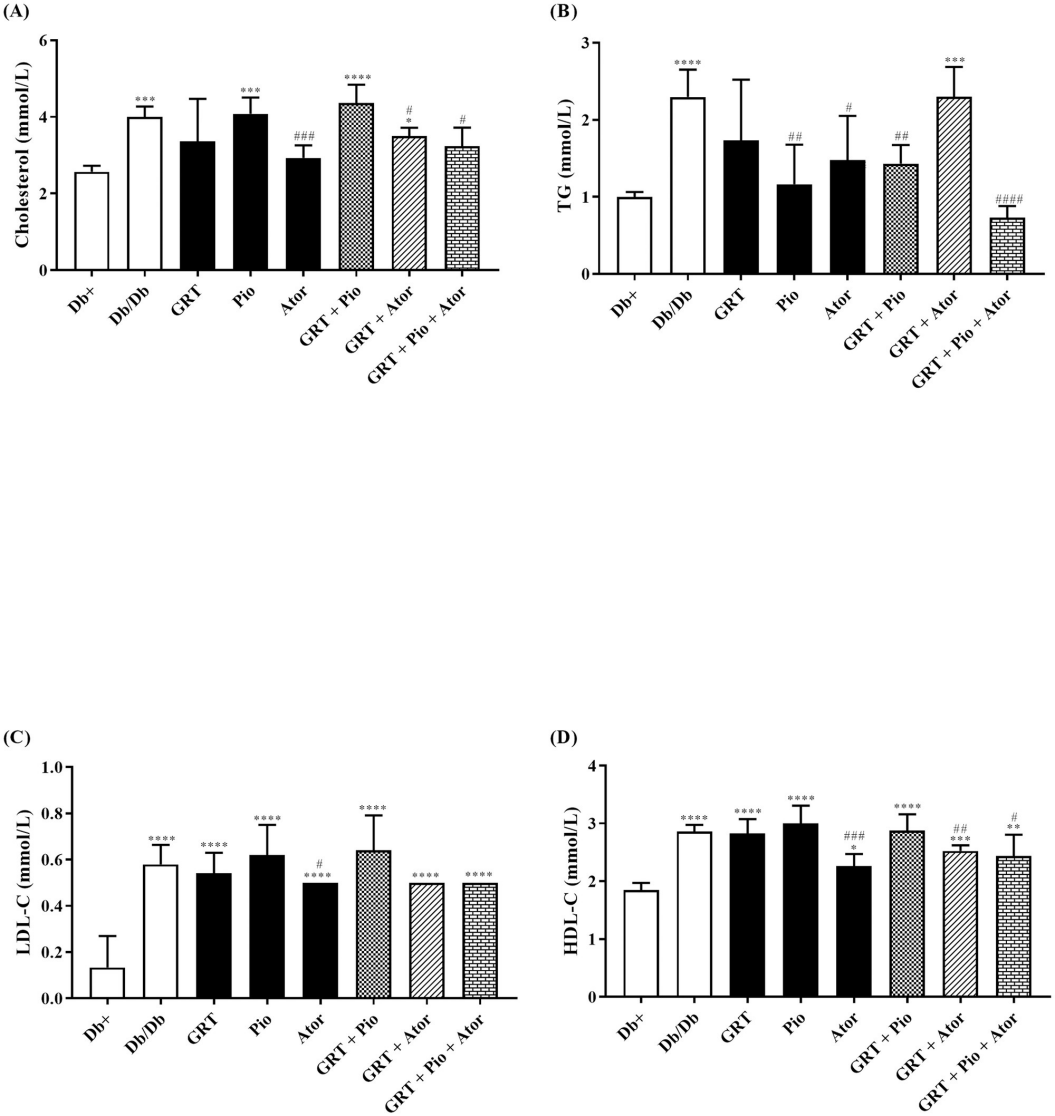
The effect of GRT (100 mg/kg BW), Pio (25 mg/kg BW), and Ator (80 mg/kg BW) mono- and co-therapies on changes in serum lipid contents. Serum lipid contents of (a) cholesterol, (b) TG, (c) LDL-C, and (d) HDL-C were measured after 5 weeks of treatment. Results are expressed as the mean ± SEM. *p < 0.05, **p < 0.01 and ****p < 0.0001 vs *db+*; ^#^p < 0.05, ^##^p < 0.01 and ^###^p < 0.0001 vs *db/db* (One-Way ANOVA followed by Dunnett/Bonferroni multiple comparison test). TG, triglyceride; LDL-C, low density lipoprotein cholesterol and HDL-C, high density lipoprotein cholesterol; GRT, green rooibos extract; Pio, pioglitazone; and Ator, atorvastatin.

Combination therapies of GRT and pioglitazone, and GRT, pioglitazone and atorvastatin showed significant increases in body and liver weights (Table 1). However, atorvastatin as well as the combination of GRT with pioglitazone, and GRT with pioglitazone and atorvastatin significantly reduced RF weights (Table 1). Following treatment, serum cholesterol, TG and LDL-C levels were significantly increased in *db/db* mice compared to the *db+* mice (Fig 2a–2d). Atorvastatin reduced cholesterol (from 4.00 ± 0.12 to 2.93 ± 0.13; p = 0.00003), TG (from 2.77 ± 0.50 to 1.48 ± 0.23; p = 0.04), and HDL-C (from 2.86 ± 0.05 to 2.27 ± 0.08; p = 0.0003) levels compared to the *db/db* mice control (Fig 2a–2d). GRT and atorvastatin co-therapy lowered cholesterol (from 4.00 ± 0.12 to 3.50 ± 0.11; p = 0.02) and HDL-C (from 2.86 ± 0.05 to 2.53 ± 0.05; p = 0.0002) levels (Fig 2a–2d). Similarly, the triple therapy combination of GRT-pioglitazone-atorvastatin decreased serum cholesterol (from 4.00 ± 0.12 to 3.24 ± 0.22; p = 0.02), TG (from 2.77 ± 0.50 to 0.73 ± 0.07; p < 0.0001), and HDL-C (from 2.86 ± 0.05 to 2.44 ± 0.16; p = 0.04) levels.

### GRT, pioglitazone, and atorvastatin co-therapy reduces hepatic steatosis

**Fig 3:**
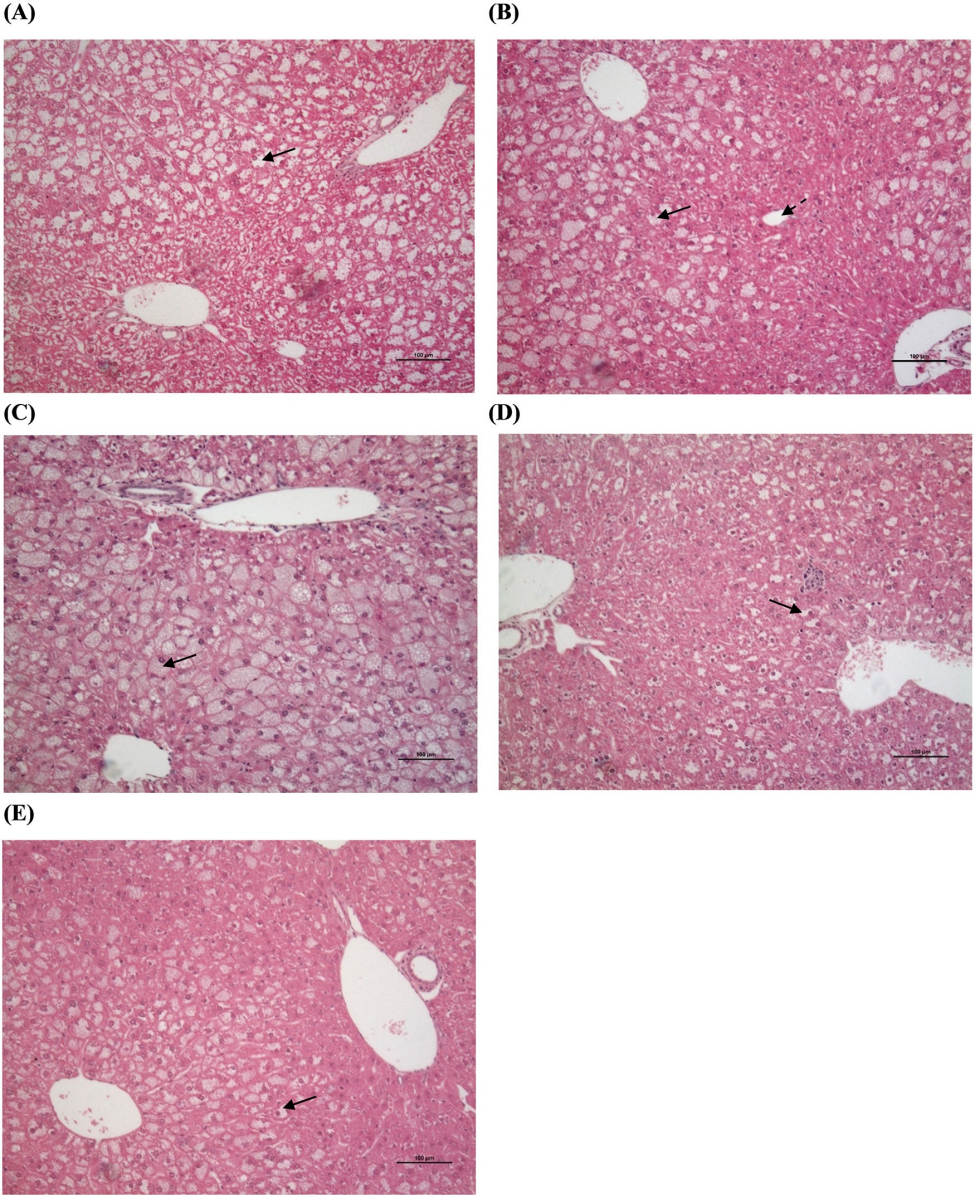
Histological assessment of H&E stained liver sections from 11-week old obese *db/db* mice treated with (a) *db/db* control, (b) GRT (100 mg/kg BW), (c) Pio (25 mg/kg BW), (d) Ator (80 mg/kg BW), and (e) GRT with Pio and Ator combination for 5 weeks. Untreated obese *db/db* control (a) mice presented with mediovesicular type steatosis across all acinar zonal areas. GRT treated obese *db/db* (b) mice presented with a mixed steatotic appearance of micro- and mediovesicular lipid accumulation predominantly in the central lobular areas. Pio treatment (c) presented with microvesicular type steatosis across acinar zonal areas. Ator treated (d) mice presented with mixed micro- and mediovesicular steatosis type present mostly in the centrilobular area. The combination of GRT, Pio and Ator (e) presented with a microvesicular type of steatosis limited to the centrilobular areas. Microvesicular steatosis represented by a bold arrow and mediovesicluar steatosis by a dotted line arrow. Magnification x 200; scale bar 100 μm. GRT, green rooibos extract; Pio, pioglitazone; and Ator, atorvastatin.

In comparison to the lean *db+* littermates, the *db/db* mice presented with an increase in micro-, mediovesicular and macrovesicular steatosis (Table 2, Fig 3). Apart from hepatic steatosis the mice had no histopathological evidence or other features of non-alcoholic steatohepatitis (NASH), which include signs of inflammation or fibrosis, were observed. Atorvastatin alone and the combination of atorvastatin with GRT reduced mediovesicular steatosis.

**Table 2:** Histological steatotic scoring.

	Treatment groups						
	*db/db*	GRT (100 mg/kg)	Pio (25 mg/kg)	Ator (80 mg/kg)	GRT (100 mg/kg) + Pio (25 mg/kg)	GRT (100 mg/kg) + Ator (80 mg/kg)	GRT (100 mg/kg) + Pio (25 mg/kg) + Ator (80 mg/kg)
**Microvesicular steatosis**	-	1	3	1	2	1	2
**Mediovesicular steatosis**	3	2	-	2	2	2	-
**Macrovesicular steatosis**	-	-	-	-	-	-	-

Results for the histological steatosis score based on Trak-Smayra *et al*. (2011) [38] and Liang *et al*. (2014) [39] are presented. Score 0: < 5%; Score 1: 5–33%; Score 2: 34–66%; Score 3: > 66%. GRT, green rooibos extract; Pio, pioglitazone; Ator, atorvastatin.

The histological scoring of steatotic severity and type in the obese *db/db* mice confirmed the predominance of mediovesicular lipid accumulation (intensity score of 3) of hepatocytes present in all acinar lobular areas. After 5 weeks of treatment, GRT slightly reduced the scoring intensity of mediovesicular lipid accumulation (intensity score of 2.2), evident as a mixed micro- and mediovesicular pattern, predominantly limited to the centrilobular and mediolobular areas. Pioglitazone treatment altered the predominant type of lipid accumulation to a microvesicular type across all lobular areas, whilst atorvastatin treatment reduced the abundance of lipid accumulation with evidence of both mixed micro- and mediovesicular steatosis mainly present in the centri- and mediolobular areas. The combination of GRT, pioglitazone and atorvastatin did not improve the intensity score of the monotherapies but reduced the appearance to a microvesicular type of lipid accumulation.

### GRT and atorvastatin co-therapy upregulates gene expression

mRNA expression of genes involved in lipid and fatty acid metabolism and lipogenesis in the liver were investigated to provide insight into the possible mechanism(s) of GRT, either as a mono- or co-therapy with pioglitazone and/or atorvastatin, and because atorvastatin is primarily metabolized by the liver.

The data are summarized in Table 3 with representative figures showed in supplementary S1a–S1g Fig. GRT monotherapy and the GRT and pioglitazone co-treatment increased *Fasn* (p = 0.02) mRNA expression in *db/db* mice. Both GRT and atorvastatin monotherapies and their co-treatment downregulated *Apoa1* mRNA expression (p < 0.05). Their co-therapy also significantly upregulated *Fasn* (p = 0.04), *Pparγ* (p = 0.004), *Pparα* (p = 0.002), and *Srebp1* (p = 0.002). Co-therapy of GRT with pioglitazone upregulated *Fasn* (p = 0.02) mRNA expression only. Furthermore, the combination of GRT, atorvastatin and pioglitazone reduced *Apoa1* (p = 0.006) and *Scd1* (p = 0.03) mRNA expression, and upregulated *Fasn* (p = 0.04), *Pparα* (p = 0.04), and *Srebp1* (p = 0.03) mRNA expression.

**Table 3:** Summary of liver gene expression in *db/db* mice.

Groups	*db/db*	GRT (100 mg/kg)	Pio (25 mg/kg)	Ator (80 mg/kg)	GRT (100 mg/kg) + Pio (25 mg/kg)	GRT (100 mg/kg) + Ator (80 mg/kg)	GRT (100 mg/kg) + Pio (25 mg/kg) + Ator (80 mg/kg)
Genes							
*Apoa1*	4.9 ± 0.5**	2.2 ± 1.0^#^	1.0 ± 0.7**	2.2 ± 1.1^#^	1.2 ± 1.0**	4.5 ± 3.4^#^	2.2 ± 0.2^##^
*Fasn*	1.8 ± 0.5	2.9 ± 2.6^#^	4.3 ± 5.9	3.1 ± 4.4	6.6 ± 7.6*^,^ ^#^	5.4 ± 6.8^#^	4.0 ± 4.5^#^
*Pparγ*	3.9 ± 0.7*	1.8 ± 1.2	1.8 ± 0.9	2.7 ± 1.1^#^	1.7 ± 0.8	5.6 ± 4.7***^,^ ^##^	2.1 ± 1.2
*Pparα*	1.4 ± 1.1	1.3 ± 1.2	1.4 ± 0.7	1.6 ± 2.6	1.3 ± 1.4	4.5 ± 4.0***^,^ ^##^	2.0 ± 1.9^#^
*Scd1*	3.2 ± 0.4	3.3 ± 8.1	1.0 ± 1.7	1.2 ± 1.5	3.2 ± 7.2	3.3 ± 10.0	3.1 ± 3.2^#^
*Srebp1*	1.4 ± 0.7	1.3 ± 0.9	1.8 ± 1.0	1.6 ± 1.9	1.2 ± 0.9	2.9 ± 1.8***^,^ ^##^	2.9 ± 3.3**^.^ ^#^
*Fabp*	1.6 ± 0.8	1.2 ± 0.9	2.4 ± 0.4*	1.5 ± 0.4*	1.4 ± 1.2	1.1 ± 0.4	1.7 ± 0.6

Results are presented as the mean ± SD (n = 6). Gene expression data normalized to β-actin and HPRT. Relative fold-change from untreated *db+* and *db/db* controls with

**p < 0.01 vs *db+*;

^#^p < 0.05,

^##^p < 0.01 and

^###^p < 0.0001 vs *db/db* (One-Way ANOVA followed by Dunnett multiple comparison test). GRT, green rooibos extract; Pio, pioglitazone; Ator, atorvastatin.

## Discussion

Treating 6-week-old obese *db/db* mice with the GRT-pioglitazone-atorvastatin combination for 5-weeks significantly improved hyperglycemia and dyslipidemia. As expected, the *db/db* mice became obese, and developed hyperglycemia and dyslipidemia. Clinically, combination therapies are generally prescribed to target both hyperglycemia and dyslipidemia to lower the risk for diabetic co-morbidities including cardiovascular disease [5, 6]. Each of these diseases are effectively treated with different medications. Hence multi-target therapy using different mechanisms of action to target disease states yield better results. However, toxicity from the combination of several drugs should not be overlooked, as the combined effect of different strategies may amplify various side effects of single drugs [40]. Hence effective pharmacological treatments to treat complex disease conditions such as diabetes are still lacking. In this study, GRT, an aspalathin-rich pharmaceutical grade rooibos extract, was combined with pioglitazone and atorvastatin, commonly prescribed hypoglycemic and lipid-lowering medications.

The obese *db/db* mouse is insulin-resistant and exhibits basal hyperinsulinemia, thus resembling human obese type 2 diabetes with peripheral insulin resistance [41]. Results obtained from this study showed that pioglitazone monotherapy did not alter either glucose or lipid levels, nor were there any significant effects on genes involved in lipid synthesis (*Apoa1*, *Fasn*, *Pparγ*, *Srebp1*) and metabolism (*Pparα*, *Scd1*, *Fabp1*). Pioglitazone was found to increase the lipid profile of diet induced obese C57BL6 mice when dosed at 25 mg/kg/day for 38 days [42]. Furthermore, an exacerbated development of fatty liver coupled with upregulation of lipid metabolism and PPAR signaling pathway genes were observed [42]. However, Ishida *et al*. (2004) [43] showed that *db/db* mice treated chronically for 6 weeks with pioglitazone (15 mg/kg) ameliorated hyperglycemia and hypertriglyceridemia, with further improvement of peripheral insulin sensitivity through *Ppary* activation. Similarly, Yeom *et al*. (2011) [36] showed that pioglitazone reduced insulin resistance via *Pparγ* activation in both lean and obese *db/db* mice. Kim *et al*. (2018) [14] confirmed that pioglitazone suppressed insulin-induced gluconeogenesis and glycogenolysis in the *db/db* mouse. The combination of GRT and pioglitazone showed an enhanced hypoglycemic effect, however, body and liver weights were increased, and *Fasn* mRNA expression upregulated in the liver. *Fasn* catalyses the synthesis of lipids required to maintain important cellular signaling and regulatory functions [44] and has been shown to control the activity of both *Pparγ* and *Pparα* [45, 46].

Atorvastatin did not affect body weight and liver weight, but reduced RF weights. Atorvastatin had no effect on FPG levels but reduced cholesterol, TG, LDL-C, and HDL-C levels. This is in accordance with studies that showed reduced TG and cholesterol levels of atorvastatin in mice when administered at either low (10 mg/kg) or high doses (80 mg/kg) [47, 48]. In the present study, combining GRT with atorvastatin did not enhance the efficacy of atorvastatin, suggesting that the triglyceridemic effect is solely due to atorvastatin. However, although the addition of pioglitazone to GRT and atorvastatin increased body and liver weights, it reduced RF weight. In addition, this combination significantly lowered FPG, cholesterol, TG, and HDL-C levels which could be attributable to an additive or synergistic effect when adding GRT and atorvastatin. LDL-r mediated cholesterol metabolism may be involved in the diabetogenic effect of statins. Statins may enhance cholesterol uptake in the liver and peripheral tissues via the upregulation of LDL-r, thereby reducing LDL-C levels in the blood. Thus, LDL-r provides a strong association between statin therapy and the risk of new onset T2DM. A study by Yu *et al*. (2018) [46] showed that short-term administration of atorvastatin only slightly affected glucose homeostasis in both normal and hyperlipidemic mice.

The histological assessment of hepatic steatosis type and severity in the obese *db/db* mice confirmed the presence of steatosis predominantly of a mediovesicular type involving all hepatic acinar zonal areas (pan-acinar steatosis) (Fig 3). Pioglitazone altered the pattern of lipid accumulation to a microvesicular type. However, the treatment did not affect the severity or zonal appearance of steatosis. Mice treated with GRT and atorvastatin presented with a mixed micro- and mediovesicular type of steatosis, with slightly improved severity and with less involvement of the periportal areas. The combination of GRT, pioglitazone and atorvastatin histologically showed an improved appearance of steatosis in terms of type and severity. The lipid accumulation was mainly in centrilobular and mid acinar zones, whilst the periportal areas appear normal (Fig 3). The zonal distribution of steatosis can be related to their relative proximity to the portal vein supplying nutrients and oxygen. Functional differences are attributed to specific zonal zones with gluconeogenesis and β-oxidation of fatty acids primarily occurring in the periportal zone (zone 1), whilst although not exclusively, glycolysis and lipogenesis occur mostly in the centrilobular zone (zone 3) [49]. In humans, the most common zonal pattern of steatosis associated with the metabolic syndrome in adults, involves the periportal tract. The improvement of the histology of the periportal zonal areas, especially following combination therapy, suggest that lipid and glucose utilization could have been enhanced by the treatment. This is consistent with other findings that *db/db* mice do not progress from NAFLD to steatohepatitis or liver fibrosis unless dietary or other insults are employed [50].

In the liver of obese rodents, several mitochondrial and metabolic adaptations such as fatty acid oxidation and oxidative stress occur. These adaptive changes may limit oxidative stress in the liver and control lipid accumulation. AMPK plays a role in the modulation of free fatty acid metabolism through the control of mitochondrial function which can be mediated through the coordinated regulation of mitochondrial bioenergetics. Altered AMPK expression is linked to the development of NAFLD. Aspalathin can target liver cells to regulate hepatic cellular metabolism and increase energy expenditure likely by modulating PI3K/AKT and AMPK signaling pathways [51]. AMPK can regulate β-oxidation by controlling the activities of ACC [52]. Malonyl-CoA content relies on ACC activity for synthesis [53]. Mice with mutations in the ACC gene were shown to have raised lipogenesis and reduced free fatty acid oxidation, leading to the progression of insulin resistance, glucose intolerance and NAFLD [54]. Furthermore, CPT-1 is required for mitochondrial free fatty acid uptake, and the downregulation in its expression can explain malfunctioning β-oxidation and obstructed energy metabolism [51].

Neither GRT nor atorvastatin as monotherapies displayed significant effects on genes involved in lipid synthesis and metabolism. However, concomitant administration of GRT with atorvastatin showed a significant upregulation of these genes. GRT in combination with atorvastatin significantly upregulated *Fasn*, *Pparγ*, *Pparα*, and *Srebp1* mRNA expression. In the liver, increased expression of *Pparγ*, *Scd1*, *Srebp*, and *Fabp1* is associated with hepatic steatosis [55]. Increased rates of fatty acid synthesis are a significant contributor of the development of hepatic steatosis, specifically in the livers of rodent models of insulin resistance. Lipogenesis related to steatosis is primarily controlled by the expression of *Srebp1* in the liver. In this study, GRT and atorvastatin co-use resulted in a notable reduction in serum cholesterol and HDL-C, and could be associated with an increased expression of *Fabp1* and *Apoa1*, inferring a possible restorative effect of *Fabp1* as previously seen in the livers of diabetic rats receiving a fish oil diet [56]. Conversely, in this study, the combined use of GRT with atorvastatin reduced the expression of *Apoa1*, whilst having no effect on *Fabp1*, corresponding with the reduction in HDL-C. *Apoa1* is the major protein component of HDL, which promotes the efflux of cholesterol and phospholipids from cells [57, 58], thus regulating lipoprotein metabolism. However, *Lepr*^*db/db*^ mice display defective catabolism for *Apoa1*, leading to increased HDL-C [59]. In rodents, *Fabp1* is upregulated by statins through the upregulation of *Pparα* and activation of *Fabp1* [60]. Furthermore, hepatocytes are susceptible to the deleterious effects of long chain fatty acids, impacting the capacity of hepatic lipid accumulation triggering inflammation, whilst lipid accumulation may be induced through over expression of *Fabp1* [61]. These results confirm that although we established moderate glucose and lipoprotein lowering biological effects of GRT and atorvastatin co-therapy, mechanistically, the increased expression of genes involved in fat accumulation and storage could enhance the hepatotoxic effects of atorvastatin. This corresponds with a previous study that showed increased plasma concentrations of atorvastatin after GRT supplementation increasing the likelihood of a herb-drug interaction [62].

Interestingly, the triple treatment regimen of pioglitazone to GRT and atorvastatin treatment moderated the overexpression of *Pparγ*, *Pparα*, *Fasn*, *and Srebp* genes involved with lipid metabolism and synthesis in the liver. Pioglitazone, an insulin sensitizer and primarily an anti-diabetic drug, is also prescribed for the treatment of non-alcoholic fatty liver disease [16]. A clinical trial showed that pioglitazone, apart from enhancing insulin sensitivity, also improved steatosis, inflammation, hepatocellular damage, and liver-enzyme levels of non-alcoholic steatohepatic patients [63]. These findings suggest that the modulatory effect on the lipid regulatory genes observed by adding pioglitazone to GRT and atorvastatin is likely due to the hepato-protective effect of pioglitazone. Although an increase in *Fasn* and *Srebp* expression was observed, the increase in *Pparα* could ameliorate this by increased β-oxidation and reducing the accumulation of triglycerides and fats in the liver.

The combination of GRT with pioglitazone and atorvastatin was shown to significantly improve glycemia, and subsequently improve hepatic steatosis in the diabetic *db/db* mouse model. However, despite these improvements, the role of increased expression of genes regulating hepatic lipid synthesis requires further investigation.

## Supporting information

S1 Fig (TIF)

S1 File Data availability. (DOCX)

S2 File Histological scoring. (DOCX)

## References

[1] Ormazabal V., Soumyalekshmi N., Omar E., Aguayo C., Salomon C., Zuñiga F. A. 2018. Association between insulin resistance and the development of cardiovascular disease. *Cardiovasc*. *Diabetol*., 17: 122. 10.1186/s12933-018-0762-4 30170598 PMC6119242

[2] Tangvarasittichai S. 2015. Oxidative stress, insulin resistance, dyslipidemia and type 2 diabetes mellitus. *World J*. *Diabetes*, 6: 456–480. 10.4239/wjd.v6.i3.456 25897356 PMC4398902

[3] Boullart A. C. I., Graaf J., Stalenhoef A. 2011. Serum triglycerides and risk of cardiovascular disease. *Biochimica et*. *biophysica acta*. 1821: 867–75. 10.1016/j.bbalip.2011.10.002 22015388

[4] Leon B. M., Maddox T. M. 2015. Diabetes and cardiovascular disease: Epidemiology, biological mechanisms, treatment recommendations and future research. *World J*. *Diabetes*, 6: 1246–1258. 10.4239/wjd.v6.i13.1246 26468341 PMC4600176

[5] Wang C. C. L., Hess C. N., Hiatt W. R., Goldfine A. B. 2016. Atherosclerotic cardiovascular disease and heart failure in type 2 diabetes–mechanisms, management, and clinical considerations. *Circulation*, 133: 2459–2502. 27297342 10.1161/CIRCULATIONAHA.116.022194 PMC4910510

[6] Steinberg H., Anderson M. S., Musliner T., Hanson M. E., Engel S. S. 2013. Management of dyslipidemia and hyperglycemia with a fixed-dose combination of sitagliptin and simvastatin. *Vasc*. *Health Risk Manage*, 9: 273. 10.2147/VHRM.S44330 23761972 PMC3673969

[7] Sugden M., Holness M. 2011. Pathophysiology of diabetic dyslipidemia: implications for atherogenesis and treatment. *Clin*. *Lipidol*., 6: 401–411.

[8] Balakumar P., Mahadevan N. 2011. Interplay between statins and PPARs in improving cardiovascular outcomes: a double-edged sword? *Br*. *J*. *Pharmacol*., 165: 373–379. 10.1111/j.1476-5381.2011.01597.x PMC3268191 21790534

[9] Thangavel N., Bratty M., Akhtar S. J., Ahsan W., Alhazmi H. A. 2017. Targeting peroxisome proliferator-activated receptors using thiazolidinediones: strategy for design of novel antidiabetic drugs. *Int*. *J*. *Med*. *Chem*. 2017: 1069718. 10.1155/2017/1069718 28656106 PMC5474549

[10] Wang Y., Jiang Y., Wang Y., Kang J., Yua T., Zhao H., et al. 2014. Inhibiton of cytochrome P450 isoenzymes and P-gp activity by multiple extracts of Huang-Lian-Jie-Du decoction. *J*. *Ethnopharmacol*., 156: 175–181. 10.1016/j.jep.2014.08.044 25219605

[11] Tyagi S., Gupta P., Saini A. S., Kaushal C., Sharma S. 2011. The peroxisome proliferator-activated receptor: A family of nuclear receptors role in various diseases. *J*. *Adv*. *Pharm*. *Tech*. *Res*., 2: 236–40. 10.4103/2231-4040.90879 22247890 PMC3255347

[12] Hanefeld M. 2007. Pioglitazone and sulfonylureas: effectively treating type 2 diabetes. *Int*. *J*. *Clin*. *Pract*., *Suppl*., 153: 20–27. 10.1111/j.1742-1241.2007.01361.x 17594390 PMC1974826

[13] Savage D. B. 2005. PPAR gamma as a metabolic regulator: insights from genomics and pharmacology. *Expert Rev*. *Mol*. *Med*., 7: 1–16. 10.1017/S1462399405008793 15673477

[14] Kim T., Lee J., Chae Y N., Jung I., Kim M. 2018. Additive effects of evogliptin in combination with pioglitazone on fasting glucose control through direct and indirect hepatic effects in diabetic mice. *Eur*. *J*. *Pharmacol*., 830: 95–104. 10.1016/j.ejphar.2018.04.033 29727638

[15] Czaja M. J. 2009. Pioglitazone: More than just an insulin sensitizer. *Hepatology*, 49: 1427–1430. 10.1002/hep.22983 19402055 PMC2689873

[16] Soran H., Dent R., Durrington P. 2017. Evidence-based goals in LDL-C reduction. *Clin*. *Res*. *Cardiol*., 106: 237–248. 10.1007/s00392-016-1069-7 28124099 PMC5360845

[17] Zhou B., Zhu L., Liu H. 2015. Effect of losartan combined with atorvastatin in treatment of essential hypertension and its influence on the plasma high sensitive C reactive protein, endothelial factors, and levels of nitric oxide. *Chin*. *J*. *Primary Med*. *Pharm*., 22: 86–88.

[18] Lella M., Indira K. 2013. A comparative study of efficacy of atorvastatin alone and its combination with fenofibrate on lipid profile in type 2 diabetes mellitus patients with hyperlipidemia. *J*. *Adv*. *Pharm*. *Technol*. *Res*., 4: 166. 10.4103/2231-4040.116778 24083205 PMC3777308

[19] Schonewille M., de Boer J. F., Mele L., Wolters H., Bloks V. W., Wolters J. C., et al. 2016. Statins increase hepatic cholesterol synthesis and stimulate fecal cholesterol elimination in mice. *J*. *Lipid Res*., 57: 1455–1464. 10.1194/jlr.M067488 27313057 PMC4959861

[20] Cai A., Zhou Y., Li L. 2015. Rho‐GTPase and atherosclerosis: pleiotropic effects of statins. *J*. *Am*. *Heart Assoc*., 4: e002113. 10.1161/JAHA.115.002113 26124206 PMC4608090

[21] Nohria A., Prsic A., Liu P. Y, Okamoto R., Creager M. A., Selwyn A., et al. 2009. Statins inhibit Rho kinase activity in patients with atherosclerosis. *Atherosclerosis*, 205: 517–521. 10.1016/j.atherosclerosis.2008.12.023 19167712 PMC2717178

[22] Low Wang C. C., Hess C. N., Hiatt W. R., Goldfine A. B. 2016. Clinical update: cardiovascular disease in diabetes mellitus: atherosclerotic cardiovascular disease and heart failure in type 2 diabetes mellitus–mechanisms, management, and clinical considerations. *Circulation*, 133: 2459–2502. 10.1161/CIRCULATIONAHA.116.022194 27297342 PMC4910510

[23] Cicero A. F. G., Colletti A., Bajraktari G., Descamps O., Djuric D.M., Ezhov M., et al. 2017. Lipid lowering nutraceuticals in clinical practice: position paper from an International Lipid Expert Panel. *Arch*. *Med*. *Sci*., 13: 965–1005. 10.5114/aoms.2017.69326 28883839 PMC5575230

[24] Fraga C. G., Galleano M., Verstraeten S. V., Oteiza P. I. 2010. Basic biochemical mechanisms behind the health benefits of polyphenols. *Mol*. *Aspects Med*., 31: 435–445. 10.1016/j.mam.2010.09.006 20854840

[25] Kawano A., Nakamura H., Hata S., Minakawa M., Miura Y., Yagasaki K. 2009. Hypoglycemic effect of aspalathin, a rooibos tea component from *Aspalathus linearis*, in type 2 diabetic model db/db mice. *Phytomedicine*, 16: 437–443. 10.1016/j.phymed.2008.11.009 19188054

[26] Muller C. J., Joubert E., de Beer D., Sanderson M., Malherbe C. J., Fey S. J., et al. 2012. Acute assessment of an aspalathin-enriched green rooibos (*Aspalathus linearis*) extract with hypoglycemic potential. *Phytomedicine*, 20: 32–39. 10.1016/j.phymed.2012.09.010 23083813

[27] Kamakura R., Son M. J., de Beer D., Joubert E., Miura Y., Yagasaki K. 2015. Antidiabetic effect of green rooibos (*Aspalathus linearis*) extract in cultured cells and type 2 diabetic model KK-A(y) mice. *Cytotechnology*, 67: 699–710. 10.1007/s10616-014-9816-y 25410530 PMC4474990

[28] Johnson R., de Beer D., Dludla P. V., Ferreira D., Muller C. J. F., Joubert E. 2018. Aspalathin from rooibos (*Aspalathus linearis*): A bioactive C-glucosyl dihydrochalcone with potential to target the metabolic syndrome. *Planta Med*., 84: 568–583. 10.1055/s-0044-100622 29388183

[29] Beltrán-Debón R., Rulla A., Rodríguez-Sanabriaa F., Iswaldi I., Herranz-López M., Aragonèsa G., et al. 2011. Continuous administration of polyphenols from aqueous rooibos (Aspalathus linearis) extract ameliorates dietary-induced metabolic disturbances in hyperlipidemic mice. *Phytomedicine*, 18: 414–424. 10.1016/j.phymed.2010.11.008 21211952

[30] Orlando P., Chellan N., Louw J., Tiano L., Cirilli I., Dludla P., et al. 2019. Aspalathin-rich green rooibos extract lowers LDL-cholesterol and oxidative status in high-fat diet-induced diabetic vervet monkeys. *Molecules*, 24: 1713. 10.3390/molecules24091713 31052590 PMC6539440

[31] Smith C., Swart A. 2018. *Aspalathus linearis* (Rooibos)–a functional food targeting cardiovascular disease. *R*. *Soc*. *Chem*., 9: 5041–5058. 10.1039/c8fo01010b 30183052

[32] Johnson R., Dludla P., Joubert E., February F., Mazibuko S., Ghoor S., et al. 2016. Aspalathin, a dihydrochalcone C-glucoside, protects H9c2 cardiomyocytes against high glucose induced shifts in substrate preference and apoptosis. *Mol*. *Nutr*. *Food Res*., 00: 1–13. 10.1002/mnfr.201500656 26773306

[33] Dludla P. V., Joubert E., Muller C. J. F., Louw J., Johnson R. 2017. Hyperglycemia-induced oxidative stress and heart disease-cardioprotective effects of rooibos flavonoids and phenylpyruvic acid-2-O-β-D-glucoside. *Nutr*. *Metab*., 14: 45. 10.1186/s12986-017-0200-8 28702068 PMC5504778

[34] Dludla P. V., Muller C. J. F., Louw J., Joubert E., Salie R., Opoku A. R., et al. 2013. The cardioprotective effect of an aqueous extract of fermented rooibos (*Aspalathus linearis*) on cultured cardiomyocytes derived from diabetic rats. *Phytomedicine*, 21: 595–601. 10.1016/j.phymed.2013.10.029 24268738

[35] Ascierto P. A., Marincola F. M. 2011. Combination therapy: the next opportunity and challenge of medicine. *J*. *Transl*. *Med*., 9: 115. 10.1186/1479-5876-9-115 21777474 PMC3157452

[36] Yeom J., Kim E. S., Park H., Ham D., Sun G., Kim J., et al. 2011. Both sitagliptin analogue & pioglitazone preserve the β-cell proportion in the islets with different mechanism in non-obese and obese diabetic mice. *BMB Rep*., 44: 713–718. 10.5483/BMBRep.2011.44.11.713 22118536

[37] Patel O., Muller C., Joubert E., Louw J., Rosenkranz B., Awortwe C. 2016. Inhibitory interactions of *Aspalathus linearis* (rooibos) extracts and compounds, aspalathin and Z-2-(β-D-Glucopyranosyloxy)-3-phenylpropenoic acid, on cytochromes metabolizing hypoglycemic and hypolipidemic drugs. *Molecules*, 21. 10.3390/molecules21111515 27845750 PMC6273468

[38] Trak-Smayra V., Paradis V., Massart J., Nasser S., Jebara V., Fromenty B. 2011. Pathology of the liver in obese and diabetic ob⁄ob and db⁄db mice fed a standard or high-calorie diet. *Int*. *J*. *Exp*. *Path*., 92: 413–421. 10.1111/j.1365-2613.2011.00793.x 22118645 PMC3248077

[39] Liang W., Menke A. L., Driessen A., Koek G. H., Lindeman J. H., Stoop R., et al. 2014. Establishment of a general NAFLD scoring system for rodent models and comparison to human liver pathology. *PLoS ONE* 9: e115922. 10.1371/journal.pone.0115922 25535951 PMC4275274

[40] Jia C., Huan Y., Liu S., Hou S., Sun S., Li C., et al. 2015. Effect of Chronic Pioglitazone treatment on hepatic gene expression profile in obese C57BL/6J mice. *Int*. *J*. *Mol*. *Sci*., 16: 12213–12229. 10.3390/ijms160612213 26035752 PMC4490440

[41] Wang B., Chandrasekera P. C., Pippin J. J. 2014. Leptin- and leptin receptor-deficient rodent models: relevance for human type 2 diabetes. *Curr*. *Diabetes Rev*., 10: 131–145. 10.2174/1573399810666140508121012 24809394 PMC4082168

[42] Solinas G., Borén J., Dulloo A. G. 2015. De novo lipogenesis in metabolic homeostasis: More friend than foe? *Mol*. *Metab*., 4: 367–377. 10.1016/j.molmet.2015.03.004 25973385 PMC4421107

[43] Ishida H., Takizawa M., Ozawa S., Nakamichi Y., Yamaguchi S., Katsuta H., et al. 2004. Pioglitazone improves insulin secretory capacity and prevents the loss of beta-cell mass in obese diabetic db/db mice: Possible protection of beta cells from oxidative stress. *Metabolism*, 53: 488–494. 10.1016/j.metabol.2003.11.021 15045697

[44] Wu W., Zhao L., Yang P., Zhou W., Li B., Moorhead J. F., et al. 2016. Inflammatory stress sensitizes the liver to atorvastatin-induced injury in ApoE-/- mice. *PLoS One*, 11: e0159512. 10.1371/journal.pone.0159512 27428373 PMC4948878

[45] Zhang N., Huan Y., Huang H., Song G. M., Sun S. J., Shen Z. F. 2010. Atorvastatin improves insulin sensitivity in mice with obesity induced by monosodium glutamate. *Acta Pharmacol*. *Sin*., 31: 35–42. 10.1038/aps.2009.176 20023693 PMC4002692

[46] Yu Q., Wang F., Meng X., Gong Y., Wang Y., Xu C., et al. 2018. Short‑term use of atorvastatin affects glucose homeostasis and suppresses the expression of LDL receptors in the pancreas of mice. *Mol*. *Med*. *Rep*., 18: 2780–2788. 10.3892/mmr.2018.9239 30015940 PMC6102652

[47] Jiang H., Zheng H. 2019. Efficacy and adverse reaction to different doses of atorvastatin in the treatment of type II diabetes mellitus. *Biosci*. *Rep*., 39: BSR20182371. 10.1042/BSR20182371 31189741 PMC6609551

[48] Chalasani N., Fontana R. J., Bonkovsky H. L., Watkins P. B., Davern T., Serrano J., et al. 2008. Causes, clinical features, and outcomes from a prospective study of drug-induced liver injury in the United States. *Gastroenterology*, 135: 1924–1934. 10.1053/j.gastro.2008.09.011 18955056 PMC3654244

[49] Hall Z., Bond N. J., Ashmore T., Sanders F., Ament Z., Wang X., et al. 2017. Lipid zonation and phospholipid remodeling in nonalcoholic fatty liver disease. *Hepatology*, 65: 1165–1180. 10.1002/hep.28953 27863448 PMC5396354

[50] Devarshi P. P., Jangale N. M., Ghule A. E., Bodhankar S. L., Harsulkar A. M. 2013. Beneficial effects of flaxseed oil and fish oil diet are through modulation of different hepatic genes involved in lipid metabolism in streptozotocin–nicotinamide induced diabetic rats. *Genes Nutr*., 8: 329–342. 10.1007/s12263-012-0326-2 23225194 PMC3639321

[51] Mazibuko-Mbeje S. E., Dludla P. V., Roux C., Johnson R., Ghoor S., Joubert E., et al. 2019. Aspalathin-enriched green rooibos extract reduces hepatic insulin resistance by modulating PI3K/AKT and AMPK pathways. *Int*. *J*. *Mol*. *Sci*., 20: 633. 10.3390/ijms20030633 30717198 PMC6387445

[52] Watt M. J., Steinberg G. R., Chen Z. P., Kemp B. E., Febbraio M. A. 2006. Fatty acids stimulate AMP-activated protein kinase and enhance fatty acid oxidation in L6 myotubes. *J*. *Physiol*., 574: 139–147. 10.1113/jphysiol.2006.107318 16644805 PMC1817791

[53] Li Y. C., Qiao J. Y., Wang B. Y., Bai M., Shen J. D., Cheng Y. X. 2018. Paeoniflorin ameliorates fructose-induced insulin resistance and hepatic steatosis by activating LKB1/AMPK and AKT pathways. *Nutrients*, 10: 102. 10.3390/nu10081024 30081580 PMC6116094

[54] Fullerton M. D., Galic S., Marcinko K., Sikkema S., Pulinilkunnil T., Chen Z. P., et al. 2013. Single phosphorylation sites in Acc1 and Acc2 regulate lipid homeostasis and the insulin-sensitizing effects of metformin. *Nat*. *Med*., 19: 1649–1654. 10.1038/nm.3372 24185692 PMC4965268

[55] Chan D. C., Watts G. F. 2006. Apolipoproteins as markers and managers of coronary risk. *J*. *Assoc*. *Physicians*, 99: 277–287. 10.1093/qjmed/hcl027 16504986

[56] Silver D. L., Jiang X. C., Tall A. R. 1999. Increased high density lipoprotein (HDL), defective hepatic catabolism of ApoA-I and ApoA-II, and decreased ApoA-I mRNA in ob/ob mice. Possible role of leptin in stimulation of HDL turnover. *J Biol Chem*., 274: 4140–4146. 10.1074/jbc.274.7.4140 9933608

[57] Landrier J. F., Thomas C., Grober J., Duez H., Percevault F., Souidi M., et al. 2004. Statin induction of liver fatty acid-binding protein (L-FABP) gene expression is peroxisome proliferator-activated receptor-α-dependent. *J*. *Biol*. *Chem*., 279: 45512–45518. 10.1074/jbc.M407461200 15337740

[58] Tamang H. K., Timilsina U., Singh K. P., Shrestha S., Raman R. K., Panta P., et al. 2014. Apo B/Apo AI ratio is statistically a better predictor of cardiovascular disease (CVD) than conventional lipid profile: a study from Kathmandu Valley, Nepal. *J*. *Clin*. *Diagn*. *Res*., 8: 34. 10.7860/JCDR/2014/7588.4000 24701475 PMC3972591

[59] Johnson R., Dludla P. V., Muller C. J. F., Huisamen B., Essop M. F., Louw J. 2017. The transcription profile unveils the cardio-protective effect of aspalathin against lipid toxicity in an in vitro H9c2 model. *Molecules*, 22: 219; 10.3390/molecules22020219 28146135 PMC6155936

[60] Wang G., Bonkovsky H. L., de Lemos A., Burczynski F. J. 2015. Recent insights into the biological functions of liver fatty acid binding protein 1. *J*. *Lipid Res*., 56: 2238–2247. 10.1194/jlr.R056705 26443794 PMC4655993

[61] Sanyal A. J., Chalasani N., Kowdley K. V., McCullough A., Diehl A. M., Bass N. M., et al. 2010. Pioglitazone, vitamin E, or placebo for nonalcoholic steatohepatitis. *N*. *Engl*. *J*. *Med*., 362: 1675–1685. 10.1056/NEJMoa0907929 20427778 PMC2928471

[62] Patel O., Muller C. J. F., Joubert E., Rosenkranz B., Taylor M. J. C., Louw J., et al. 2019. Pharmacokinetic interaction of green rooibos extract with atorvastatin and metformin in rats. *Front*. *Pharmacol*., 10: 1243. 10.3389/fphar.2019.01243 31708777 PMC6822546

[63] Athyros V. G., Alexandrides T. K., Bilianou H., Cholongitas E., Doumas M., Ganotakis E. S., et al. 2017. The use of statins alone, or in combination withpioglitazone and other drugs, for the treatment ofnon-alcoholic fatty liver disease/non-alcoholicsteatohepatitis and related cardiovascular risk. An expert panel statement. *Metab*., *Clin*. *Exp*., 71: 17–32. 28521870 10.1016/j.metabol.2017.02.014

