# Supplementary figures and images for: Therapeutic effects of an aspalathin-rich green rooibos extract, pioglitazone and atorvastatin combination therapy in diabetic db/db mice
Source: PLoS One. 2021 May 13;16(5):e0251069. doi: 10.1371/journal.pone.0251069 (PMC8118332; doi:10.1371/journal.pone.0251069)

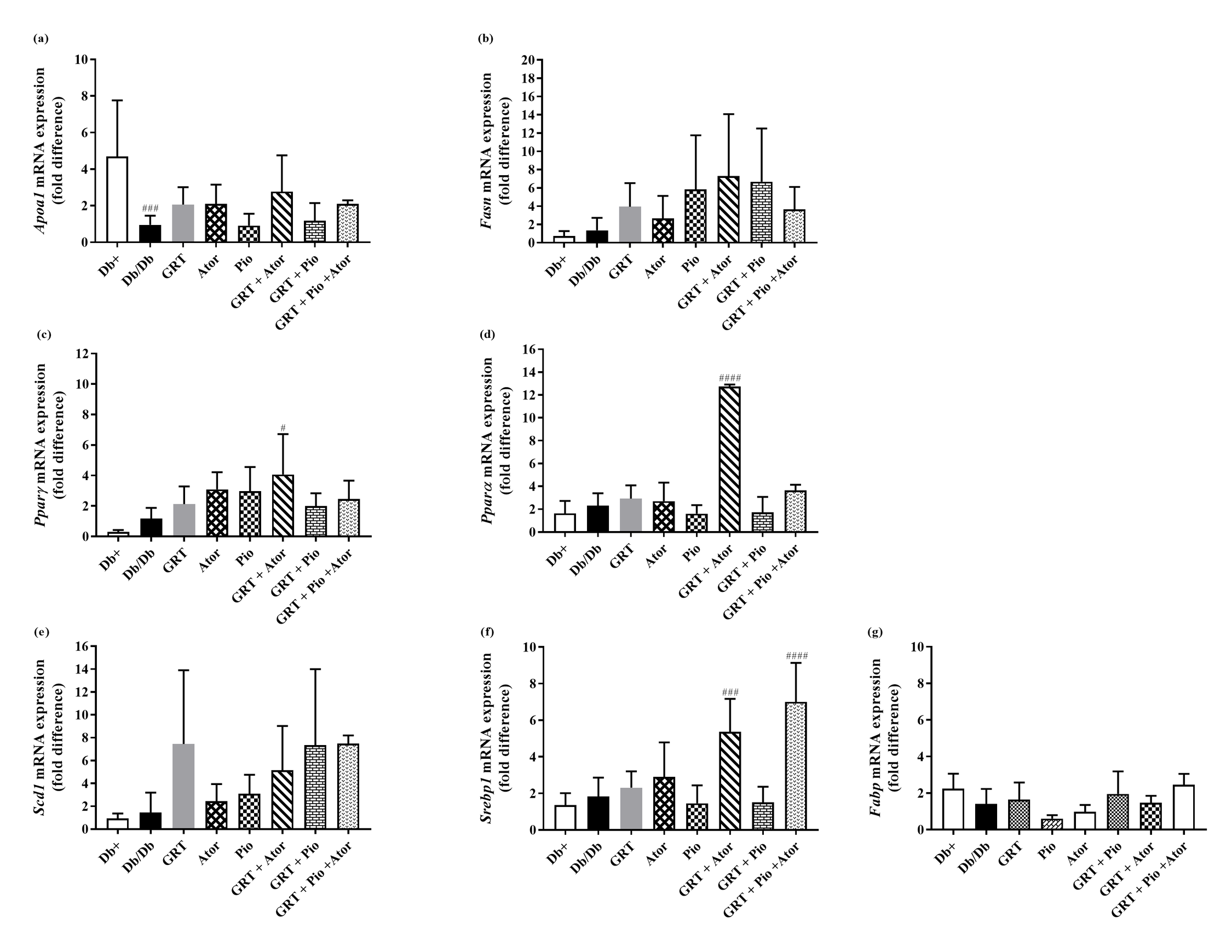

Supplement: S1 Fig — (TIF) [file pone.0251069.s001.tif]
