# Supplementary material for: Therapeutic effects of an aspalathin-rich green rooibos extract, pioglitazone and atorvastatin combination therapy in diabetic db/db mice
Source: PLoS One. 2021 May 13;16(5):e0251069. doi: 10.1371/journal.pone.0251069 (PMC8118332; doi:10.1371/journal.pone.0251069)
Supplement: S1 File — (DOCX) [file pone.0251069.s002.docx]

**Fasting blood glucose**

**Body weights**

**IPGTT**

**Liver and RF weights**

**Lipogram data**
